# Implementation of a coaching training for enhancing empathy and emotional intelligence skills in health science students: a prospective study

**DOI:** 10.1186/s12909-024-05076-z

**Published:** 2024-01-22

**Authors:** María Encarnación Aguilar-Ferrándiz, Sonia Toledano-Moreno, Antonio Casas-Barragán, Manuel Albornoz-Cabello, Rosa María Tapia-Haro, María Correa-Rodríguez

**Affiliations:** 1 Department of Physical Therapy, Faculty of Health Sciences, University of Granada (UGR), Instituto de Investigación Biosanitaria ibs.GRANADA. Granada, Ave. de la Ilustración, 60, 18071 Granada, Spain; 2 Department of Physical Therapy, Faculty of Nursing Physiotherapy and Podiatry, University of Sevilla (US), Sevilla, Spain; 3 Department of Nursing, Faculty of Health Sciences, University of Granada (UGR), Instituto de Investigación Biosanitaria ibs.GRANADA, Granada, Spain

**Keywords:** Academic coaching, Empathy, Emotional intelligence, Skills, Undergraduate students

## Abstract

**Background:**

Empathy and emotional intelligence are core competencies in the educational curriculum of health science students, both play a significant role in teamwork relationships and in attention patient’s cares; so innovative strategies to enhance these emotional skills are required. We prospectively tested an academic coaching program for improving empathy and emotional intelligence in students of health sciences degrees.

**Methods:**

A prospectively single arm intervention study was performed in undergraduate students of nursing, physiotherapy and occupational therapy of the Faculty of Health Sciences from the University of Granada (Spain). The three groups of students participated in nine sessions of coaching, which included a training program to manage patient’s priorities and communication, adherence to treatment, motivation and satisfaction. Survey data included the Cognitive and Affective Empathy Test (TECA), the Trait Meta-Mood Scale (TMMS-24) and the Interpersonal Reactivity Index (IRI) which were assessed at baseline and post-intervention.

**Results:**

A total of 93 students of 259 (mean age of 21.6 ± 3.2 years) participated in the study and completed the sessions of coaching/surveys. After the intervention, we observed an improvement in the cognitive dimension of empathy among nursing students (*p* = 0.035) and in the affective dimension of empathy in physiotherapy students (*p* = 0.044). In addition, an increase on perceived emotional intelligence among students was achieved only in nursing/physiotherapy groups (*p* ≤ 0.048). Finally, slight improvements were founded in the dimensions “Perspective-Taking” and “Personal Distress” of the occupational therapy group (*p* ≤ 0.031). No significant differences were found for the rest of variables of TECA (*p* ≥ 0.052), TMMS-24 (*p* ≥ 0.06) and IRI (*p* ≥ 0.12).

**Conclusions:**

This study shows that an academic coaching intervention with students from health sciences degrees improves their empathy skills and self-perceived emotional intelligence. The current findings can be used to determine more effective approaches to implementing academic coaching interventions based in better designs as clinical trial studies.

## Background

The educational process for university degrees in Medicine and Health Sciences, is based on the development of specific knowledge and a set of common, transversal learning skills, that are acquired through the study programme [1]. In this line, empathy and emotional intelligent (EI) have been previous described as transversal core skills for health sciences student’s curriculum since they might affect the ability to communicate effectively with patients and their team [2, 3]. The care work involved in health sciences disciplines is closely related to caring for patients who are suffering, to a greater or lesser extent, from disabilities, vulnerabilities, or comorbidity [4]. Consequently, healthcare professionals must be able to understand the patient’s situation in order to improve their adherence to treatment and the quality of the care they receive [5]. As a result, the acquisition and development of empathy and EI skills, is particularly important for patient interaction at the clinical setting in nursing, physiotherapy and occupational therapy disciplines [6].

Different studies [1, 7, 8] have demonstrated that empathic behaviour of healthcare professionals is a fundamental aspect of any therapeutic relationship: not only increases the subjective satisfaction of patients, also improves the diagnostic and recovery processes, as well as adherence to - and the outcomes of - treatment [9]. In turn, all of this translates to a reduction in the use of resources, with consequent savings in healthcare costs and increased well-being on the part of professionals [10]. Therefore, the empathic capacity and EI of healthcare professionals play a crucial role in dealing with processes related to health, disease and therapy, and are skills that can be taught and refined through education and practice [10, 11]. However, although it is recognised that these skills must be acquired during the process of training professionals in the health sciences field [1, 10], traditionally these transversal skills have been developed on a self-taught basis through professional practice and daily contact with patients, and are not often included in the specific curricula of the aforementioned university programmes [1].

In this context, the most recent literature agrees that Coaching is an innovative teaching methodology for training professionals in the health sciences [12–15]. Coaching was first used in areas such as sports and business, where its effectiveness is proven and represents a move away from traditional, instruction-based learning methods and towards self-directed learning, which is considered more appropriate [15, 16]. It enables educators to work with students on relevant aspects related to EI and empathic behaviour and allows trainers to help students tackle professional and career-related challenge [8, 17–19]. Coaching is based on the principles of the Knowles’ adult learning framework [14, 20], their methodologies are student-centred and based on the Socratic method and the concept of existentialism include asking reflective questions [20]. Students become active agents in their own academic objectives through a constant process of action-reflection-action, in dialogue with a qualified partner, following a process of observation, reflection and exploration of challenges which leads to transformative learning experiences [10, 12, 14, 20, 21].

Thus, coaching can be used in the educational process to help learners improve their own self-monitoring, personal development, leadership and self-efficacy, since it helps students to deep in their emotions, thoughts and behaviours, promoting self-awareness and EI. This methodology also allows work on stress management, conflict resolution and improvement of communication skills. Overall, coaching provides a non-judgmental, supportive environment for students to explore their potential, address obstacles, and embrace positive change [16, 21]. Despite the aforementioned proprieties of coaching, the literature about the use of this academic methods in health sciences education is still scarce and, in our concern, there is not studies that analyse specific measures of empathy an EI for testing its effects in academic degree.

With this background, our study aims to draw attention to and address an insufficiency in students of health sciences degrees with regard to the transversal skills of empathy and EI, which are necessary in order to care for patients during supervised practical work. Thus, the overall purpose of this study was to explore whether an academic coaching program could improve empathic accuracy and emotional intelligence in students of health sciences degrees.

## Methods

### Design

We conducted a prospectively single arm intervention study with pre-post evaluation for assessing the effectiveness of an academic coaching programme on empathy and EI skills, in health sciences students from the Faculty of Health Sciences at the University of Granada (Spain).

### Participants and setting

Participants were students of the physiotherapy, occupational therapy and nursing degree programmes and were chosen by convenience sampling.

The study was conducted between September 2018 and July 2019 which was previously approved by Unit of quality, teaching innovation and prospective of the University of Granada. As for the ethical aspects of the procedure, students across all years of the three-degree programmes were given information regarding the purpose of the study and the right to decline at any time, all of those who voluntarily wished to participate and signed the written informed consent were included. The inclusion criteria were: Students could not have received previous training in coaching; and they had to sign the informed consent form in order to participate.

**Fig. 1:**
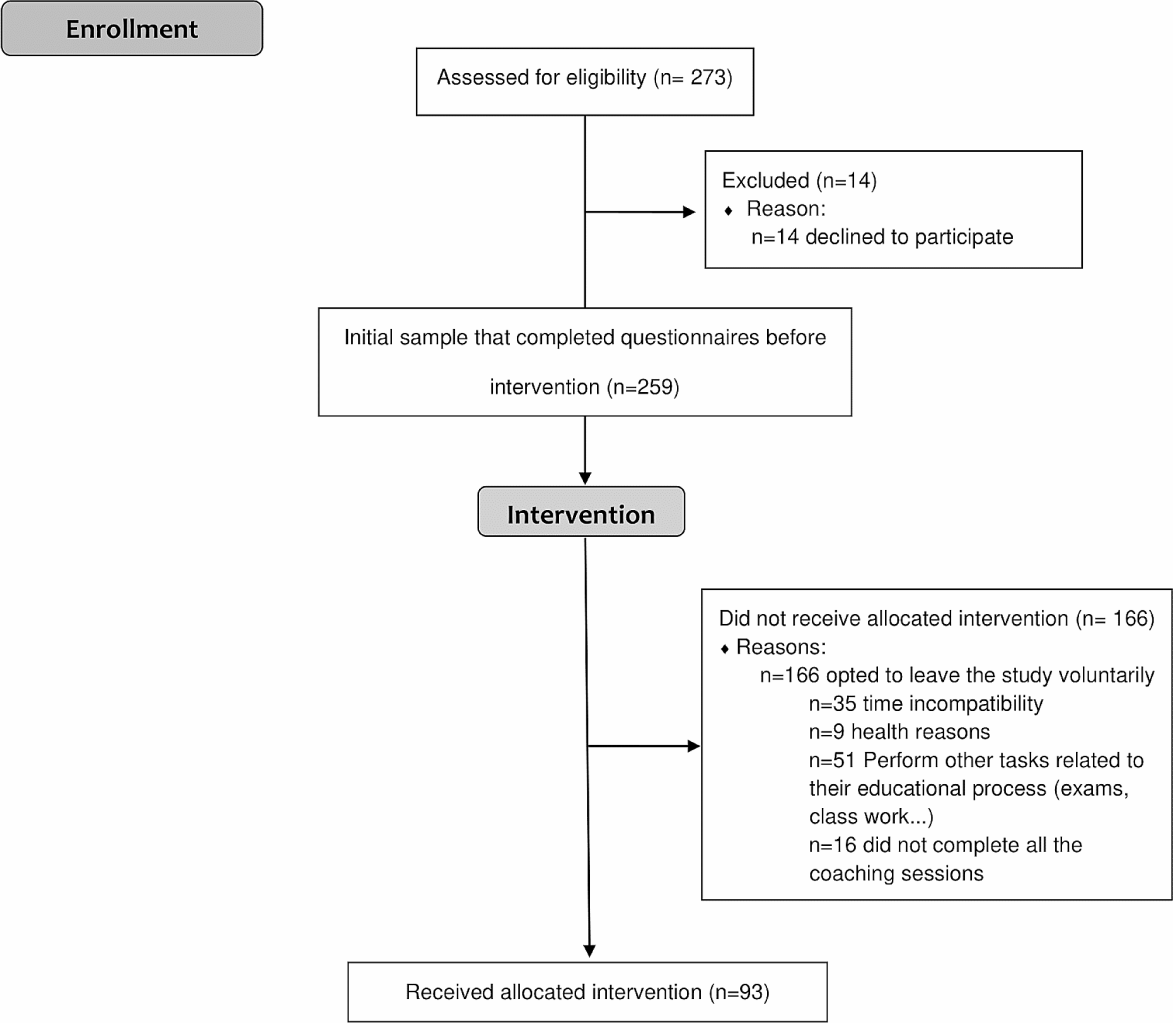
Flow diagram of the participants throughout the study

Of the 273 students who met the inclusion criteria, a total of 256 participants conformed the initial sample. Subsequently, 116 students decided to leave voluntarily the study for different reasons, so a total of 93 students who completed the intervention by attending all coaching sessions and surveys were included in the final analysis (29 in nursing group, 25 in physical therapy (physiotherapy) group and 39 in occupational therapy group). A flow diagram of the participants throughout the study is provided in Fig. 1.

### Assessments

First, all of the participants provided sociodemographic information (i.e. age, gender, degree programme, previous studies and year of study) and, via the virtual platform Lime Survey from the University of Granada, completed a series of questionnaires designed to measure their levels of empathy and EI. We used the same approach to collect information after the coaching intervention. We assessed the aforementioned skills using the following standardised questionnaires.

#### Cognitive and affective empathy test (TECA)

TECA provided an overall average score for empathy from a cognitive and affective dimension. The Spanish version consists of 33 items divided into two dimensions and four subscales. The cognitive dimension was assessed via the subscales Perspective Adoption and Emotional Understanding, while the affective dimension was assessed via the subscales Empathic Stress and Empathic Joy. Participants indicated in a Likert five-point scale (1 = strongly disagree and 5 = strongly agree) the strength of their agreement with the statements. This scale boasts adequate psychometric properties and offers a high degree of reliability and internal consistency (*r* = 0.86 and α = 0.86). Higher scores mean higher empathy levels [22].

#### Trait meta-mood scale (TMMS-24)

To assess individuals’ beliefs regarding their own EI, participants completed the TMMS-24. This scale examines three particular aspects of perceived EI: Emotional Attention, Emotional Clarity and Emotional Repair. Consist of 24 items, with eight items for each subscale. Using a Likert five-point scale (1 = strongly disagree and 5 = strongly agree), participants indicate the strength of their agreement with each of the items. The Spanish version of the TMMS presented appropriate psychometric properties with a high degree of reliability (Emotional Attention (0.90); Emotional Clarity (0.90) and Emotional Repair (0.86)) and internal consistency on the part of the subscales and an adequate test-retest correlation: Emotional Attention (*r* = 0.60), Emotional Clarity (*r* = 0.70) and Emotional Repair (*r* = 0.33). Scores between 22 and 35 have been defined as normal for attention and a range of 24–35 for clarity and repair [23].

#### Interpersonal reactivity index (IRI)

The IRI scale assesses the overall degree of empathy through the use of 28 items, with seven items per subscale, measures the cognitive aspects and emotional reaction of an individual when adopts an empathic position. The subscales that measure cognitive processes are Perspective-Taking and Fantasy, while the affective components of empathy are measured via the subscales Empathic Concern and Personal Distress. Subjects have to state their opinion regarding a series of affirmations related to their thoughts and feelings in a variety of situations in a five-point Likert scale, where 0 = Does not describe me well and 4 = Describes me very well. With regard to reliability, the alpha values ranged from 0.56 to 0.70 for the total sample, while the results regarding internal consistency of the subscales ranged from 0.68 to 0.78. Mean scores between 16.78 and 17.96 have been defined as normal for perspective-taking, a range of 15-18.75 for fantasy, a range of 19, 4–21, 67 for Empathic Concern and a range of 9.46–12.28 for Personal Distress [24].

### Intervention

We conducted an academic coaching program which was based in 9 sessions of reduced coaching group with the students, with the aim of developing clinical case studies and strengthening their emotional skills, empathy and EI through the use of discussion forums. Sessions were conducted by teaching staff from the three-degree programmes of the Faculty of Health Sciences of the University of Granada. In order to develop this methodology, during the previous academic year (2017–2018), the teaching staff received specific training on various academic coaching techniques and empathic behaviour analysis. The coaching training was provided by a professional coach, with clinically trained and more than 20 years of experience in educational coaching.

The coaching sessions were focused on the development of empathic behaviour wits were randomly assigned into a total of 6 intervention groups (3 groups of 15 participants and 3 groups of 16 participants), random assignment was geneh a view to caring for patients within different clinical contexts. The intervention protocol was designed around the following methodological activities based on educational coaching: clinical cases and identification of needs, solution proposals, and presentation of the resolution of clinical cases [16]. Participantrated using computerized randomization software that assigned each participant to their intervention group. We implement 9 group coaching sessions, once a week, with an average duration of 60 min. First, three sessions where coaches presented the clinical cases and the students had to identify the patients’ needs were performed. Subsequently, three sessions were held where, based on the needs that the students had identified in the clinical case, and taking into account the transversal knowledge they had developed, they had to propose solutions with an action plan. Finally, three sessions were carried out where the students presented their proposals for action to the group, answered questions from their classmates, and debated the proposals. Additionally, we have included four control variables that can influence the development of the coaching program: the coaching medium, the number of coaching sessions, the duration of the coaching program, and the demographics of the coaches (age) [25]. The medium of coaching was decided during the design of the study, our coaching program was based on the principles of the Knowles’ adult learning framework [10, 14, 16, 20]; the number of sessions was provided by the coaches at the end of the intervention [15, 26]; the duration of the coaching was calculated as the difference between the dates of the pre- and post-coaching surveys measured in months [15, 25, 26]; the age of the coaches was provided by the coaches at the beginning of the study (10.14,15,26). Table 1 showed a detailed overview of the coaching program and control variables.

**Table 1:** Overview of the coaching program and control variables

Sesions	Type		Intervention	Goals	
- **Sesions 1–3** **Clinical case and identification of needs**	Group Format, 6 groups: - Group 1: 15 participants - Group 2: 16 participants - Group 3: 16 participants - Group 4: 15 participants - Group 5: 16 participants - Group 6: 15 participants Time of sessions: 60 min/per group Frequency of sessions: 1 session/ a week		- Coaches presented clinical cases to each group. - Guided by their teacher/coach, each group prepared an in-depth interview, which chiefly explored the limitations suffered by the patient in their daily life as a result of their illness, the psychological and emotional repercussions of said illness, changes in their social habits, their subjective perception of their current condition. - Students had previously been instructed in the ethical commitments they were required to observe and the patient filled out the informed-consent form, which assured them that their data would remain confidential. At no time were any of the patients’ identifying details disclosed. - Using all of the information gathered from the interview, the students then had to summarise the patient’s key physical, emotional and social needs	- Enhance their emotional intelligence. - Enhance their empathy. - Enabling them to handle stress. - Manage conflicts. Identifying and approaching individual’s negative thinking. - Build more meaningful connections with other. - Improve their communication skills. - Boost self-confidence and self-efficacy. - Enhance team dynamics, foster a sense of unity, and improve the overall performance and productivity of the team.	
**- Sesions 4–6** **Proposed solution**	Group Format, 6 groups: - Group 1: 15 participants - Group 2: 16 participants - Group 3: 16 participants - Group 4: 15 participants - Group 5: 16 participants - Group 6: 15 participants Time of sessions: 60 min/per group Frequency of sessions: 1 session/ a week		- Based on the needs that the students had identified in the clinical case, the participants, taking into account the transversal knowledge that they had developed, must propose an action plan. - The action plan was focused on: - Patient priorities, communication, motivation, treatment adherence and satisfaction.	- Goal Setting and Achievement: to set clear, achievable goals and create action plans to reach them. - Make decisions. - Designed a plan to meet, or minimise, the patients’ needs including motivational goal and subjective perspective from the patients. - Enhance team dynamics, foster a sense of unity, and improve the overall performance and productivity of the team. - Application of decision-making and prioritization tools. - Application of techniques learning throughout career.	
- **Sesions 7–9** **Presentation of the clinical case**	Group Format, 6 groups: - Group 1: 15 participants - Group 2: 16 participants - Group 3: 16 participants - Group 4: 15 participants - Group 5: 16 participants - Group 6: 15 participants Time of sessions: 60 min/per group Frequency of sessions: 1 session/ a week		- Students gave a presentation in their group, in which they discussed all of the aspects mentioned above. - For their presentations they could use images of the patient or original video footage, adapted in accordance with the confidentiality criteria and with the prior consent of the patient, in order to adequately illustrate their particular clinical cases. - Following this presentation of the clinical cases, they answered questions from their fellow students and the session was opened up for discussion, which was led and guided by the coach teaching staff.	- Leadership Development. - Identifying and approaching individual’s negative thinking. - Enabling them to handle stress. - Manage conflicts.	
**Control Variables**	**Value**	**Quantification**	**Theorical reason**	**References**	
**Coaching Medium**	Principles of the Knowles’ adult learning framework	Elected model	- It focuses on competency-based, learner-driven education. - Methodologies are student-centred and based on the Socratic method that include asking reflective questions. - It is based on the six principles of the adult learning process: adults are internally motivated and can self-direct their learning process, provide life experiences and knowledge to the learning experiences, tend to be objective and oriented towards the aspects relevant, are practical and they like to be respected.	- Lovell B. What do we know about coaching in medical education? A literature review. Med Educ. 2018;52(4):376 − 90. - Shorey S, Ang E, Xin Chua JY, Sun P. Coaching interventions among healthcare students in tertiary education to improve mental well-being: A mixed studies review. Nurse Educ Today. 2022; 109:105222 - Deiorio NM, Carney PA, Kahl LE, Bonura EM, Juve AM. Coaching: A new model for academic and career achievement. Med Educ Online. 2016;21(1):33,480. - Pollak KI, Gao X, Arnold RM, Arnett K, Felton S, Fairclough DL, Kutner JS. Feasibility of Using Communication Coaching to Teach Palliative Care Clinicians Motivational Interviewing. J Pain Symptom Manage. 2020;59(4):787 − 93.	
**Number of coaching sessions by groups**	9	Total number of coaching sessions received in each group at the end of the program, provided by the coaches.	- The number of coaching sessions used usually ranges between one and twelve.	- Wolff M, Hammoud M, Santen S, Deiorio N, Fix M. Coaching in undergraduate medical education: a national survey. Med Educ Online. 2020;25(1):1,699,765. - Grover S, Furnham A. Coaching as a Developmental Intervention in Organisations: A Systematic Review of Its Effectiveness and the Mechanisms Underlying It. PLoS One. 2016;11(7):e0159137.	
**Coaching duration**	2 Months	This outcome was calculated as the difference between pre- and post- coaching evaluation, measured in months	- The usual duration of coaching programs ranges between 1.3 and 12 months	- Wolff M, Hammoud M, Santen S, Deiorio N, Fix M. Coaching in undergraduate medical education: a national survey. Med Educ Online. 2020;25(1):1,699,765. - Halliwell PR, Mitchell RJ, Boyle B. Leadership effectiveness through coaching: Authentic and change-oriented leadership. PLoS One. 2023;18(12):e0294953. - Grover S, Furnham A. Coaching as a Developmental Intervention in Organisations: A Systematic Review of Its Effectiveness and the Mechanisms Underlying It. PLoS One. 2016;11(7):e0159137.	
**Coaches age (demographics)**	Age range between 27–35 years	This variable was provided by the coaches at the beginning of the study	- In coaching, coaches and students share an equal partnership, a longitudinal relationship is established between student and coach based on trust and respect. - Therefore, we selected younger teachers, with whom students could identify better. - Coaches must collaborate with students in a stimulating and imaginative way to help them optimize their potential, encouraging self-reflection and self-improvement. - Teachers staff completed prior coaching training to avoid role confusion, learn to provide detailed information and feedback, manage group dynamics, and address a variety of topics (personal, family, mental health, academic) relevant to students. - Coaches had to be available and accessible.	- Lovell B. What do we know about coaching in medical education? A literature review. Med Educ. 2018;52(4):376 − 90. - Shorey S, Ang E, Xin Chua JY, Sun P. Coaching interventions among healthcare students in tertiary education to improve mental well-being: A mixed studies review. Nurse Educ Today. 2022; 109:105222 - Wolff M, Hammoud M, Santen S, Deiorio N, Fix M. Coaching in undergraduate medical education: a national survey. Med Educ Online. 2020;25(1):1,699,765 - Grover S, Furnham A. Coaching as a Developmental Intervention in Organisations: A Systematic Review of Its Effectiveness and the Mechanisms Underlying It. PLoS One. 2016;11(7):e0159137.	

All control variables were applied homogeneously throughout the program in all included groups

### Statistical analysis

We used the SPSS© version 24.0 for Windows for data analyses We have checked the reliability of the model and validity of the hypothesis and we have studied the normal distribution of variables, residual values and linearity of variances. The normal distribution of the variables was verified using the Kolgomorov-Smirnov test for continuous variables and the Chi-square goodness-of-Fit test for categorical variables. The residuals were analysed using the residual plot to compare the observed values with the values, distribution, and trend of the residuals. Linearity was examined using bivariate scatterplots of observed versus expected residual values. Then sociodemographic variables were examined in the three groups by using analysis of variance (ANOVA) for continuous data and chi-squared tests (*χ*^2^) for categorical data. The data for continuous variables were expressed as mean ± standard deviation (SD) and for categorical variables as frequency (%). Subsequently, we evaluate the effect of the educational coaching intervention in the variables of empathy and EI for the participants in each group. For comparison analyses between pre and post intervention we calculated the changes in variables scores for each dimension and total punctuation of TECA, TMMS-24 and IRI were analysed within groups by means (95% confidential interval) of t-tests for paired samples. Finally, we calculated the magnitude of the effect size difference according to Cohen’s standardised d-index. An effect size of ≤ 0.2 indicates a negligible difference, between ≥ 0.2 and < 0.5 a small difference, between ≥ 0.5 and < 0.8 a moderate difference, and ≥ 0.8 a large difference. Regarding the level of significance, *P* < 0 0.05 was considered significant in all tests.

## Results

### Descriptive statistics

Of the 273 students initially recruited for this study, a total of 259 met the criteria for inclusion with an average age of 21.6 (SD = 3.2). The majority of the participants were women (79.5%), the occupational therapy degree provided the greatest number of participants, accounting for 42.9% of the total. The 2.26% of the students have studied a previous degree (3 students in the physiotherapy group had previously studied occupational therapy and 1 physical activity and sports sciences; 1 student in the occupational therapy group have previously studied psychology, one student of nursing had previously completed physiotherapy). In terms of year of study or course, the highest proportion (37.1%) of participant were in the second course (see Table 2).

**Table 2:** Sociodemographic characteristics of participants

Outcomes	Nursing Group (*n* = 56)	Physical Therapy Group (*n* = 92)	Ocupacional Therapy Group (*n* = 111)	Total (*n* = 259)
Age (years)	21.6 ± 3.8	21.2 ± 3.2	21.9 ± 2.8	21.6 ± 3.2
Sex				
Male	8 (14.3)	30 (32.6)	15 (13.5)	53 (20.5)
Female	48 (85.7)	62 (67.4)	96 (86.5)	206 (79.5)
Academic Degree	53 (21.6)	91 (35.5)	110(42.9)	
Course/ Year				
First	-	25 (27.2)	3 (2.7)	28(10.8)
Second	36 (69.6)	15 (16.3)	42 (37.8)	96 (37.1)
Third	17 (30.4)	19 (20.7)	40 (36)	76 (29.3)
Fourth	-	32 (34.8)	25 (22.5)	57 (22)

Data are expressed as the mean ± standard deviation (SD) for quantitative variables or frequency and (%) for qualitative outcomes

### Change scores in nursing students group for TECA, TMMS-24 and IRI

The results demonstrated a significant increase in the scores for the Perspective Adoption subscale, which corresponds to the cognitive dimension of the TECA (t = -2.21, *p* = 0.035) in nursing students’ group. The effect size was small (*r* = -0.14). No significant differences were found for the rest of the variables (t ≥ -1.11, *p* ≥ 2.77).

There was a significant increase in the total score for the TMMS-24 (t= -3.42, *p* = 0.002), as well as in the Emotional Clarity (t = -3.12, *p* = 0.003) and Emotional Repair (t = 2.29, *p* = 0.030) subscales, with a small effect size in all cases (*r* ≥ -0.15). The emotional attention item was not significant (t=-1.27, *p* = 0.21).

No significant differences were found for the IRI scores (t ≥ 1.58, *p* ≥ 0.12) (see Table 3).

**Table 3:** Pre-post coaching intervention differences, change scores in nursing students group (*n* = 29) (95% confidence interval) for TECA, TMMS-24 and IRI

Outcome	Pre-Coaching Intervention	Post-Coaching Intervention	Cohen’s d (Pre/Post)	Score Change
**TECA**				
Total	51.34 ± 13.54	53.38 ± 11.36	-0.16	-2.14 (-6.08, 1.81)
Cognitive Empathy				
Perspective Adoption	50.93 ± 11.12	54.13 ± 11.58	-0.28	-3.21 (-6.17, -0.24)*
Emotional Understanding	53.45 ± 10.12	53.41 ± 10.50	0.01	0.03 (-3.35, 3.42)
Affective Empathy				
Empathic Stress	49.65 ± 10.66	50.07 ± 8.62	-0.04	-0.41 (-2.92, 2.09)
Empathic Joy	54.62 ± 9.83	53.65 ± 10.60	0.09	0.96 (-2.33, 4.26)
**TMMS-24**				
Total	81.10 ± 15.58	87.17 ± 13.69	-0.42	-6.07 (-9.70, -2.43)*
Emotional Attention	28.30 ± 7.80	29.70 ± 5.46	-0.21	-1.40 (-3.65, 0.85)
Emotional Clarity	26.80 ± 6.48	29.47 ± 5.97	-0.43	-2.67 (-4.37, -0.96)*
Emotional Repair	26.00 ± 7.58	28.00 ± 6.00	-0.30	-2.00 (-3.79, -0.21)*
**IRI**				
Total	66.20 ± 13.26	65.73 ± 11.53	0.04	0.47 (-2.68, 3.61)
Fantasy	16.63 ± 6.43	17.67 ± 6.00	-0.16	-0.73 (-2.02, 0.56)
Perspective-Taking	18.57 ± 4.91	18.97 ± 5.01	-0.08	-0.40 (-1.88, -1.07)
Empathic Concern	20.03 ± 4.73	19.13 ± 4.61	0.19	0.90 (-0.30, 2.10)
Personal Distress	10.97 ± 4.18	10.27 ± 4.02	0.17	0.70 (-1.03, 2.43)

TECA: Cognitive and Affective Empathy Test; TMMS-24: Trait Meta-Mood Scale; IRI: Interpersonal Reactivity Index

Values are expressed as means ± standard deviation (SD) for pre-post coaching intervention and as mean (95% confidence interval) for within-group (baseline to follow-up) and between-group change scores (at follow-up)

*Significant Student t-test, *P* < 0.05

### Change scores in physical therapy students group for TECA, TMMS-24 and IRI

In the physiotherapy students group the results demonstrated a significant increase in the scores for the Empathic Joy subscale, which corresponds to the emotional dimension of the TECA (t = -2.12, *p* = 0.044). The effect size was small (*r* = -0.12). No significant differences were found for the rest of the variables in this test (t ≥ -1.69, *p* ≥ 0.10).

There was a significant increase in the total score for the TMMS-24 (t = -2.81, *p* = 0.010) and the score for the Emotional Clarity subscale (t = -2.09, *p* = 0.048). The effect size for these scores was small (*r* ≥ -0.18). No significant differences were found in the others items of this test (t ≥-2,00, *p* ≥ 0.06). No significant differences were found for the IRI scores (t ≥ -0.16, *p* ≥ 0.12) (see Table 4).

**Table 4:** Pre-post coaching intervention differences, change scores in physical therapy students group (*n* = 25) (95% confidence interval) for TECA, TMMS-24 and IRI

Outcome	Pre-Coaching Intervention	Post-Coaching Intervention	Cohen’s d (Pre/Post)	Score Change
**TECA**				
Total	56.92 ± 8.31	58.80 ± 8.92	-0.22	-1.88 (-4.48, 0.73)
Cognitive Empathy				
Perspective Adoption	58.87 ± 9.97	59.29 ± 10.16	-0.04	-0.42(-3.56, 2.73)
Emotional Understanding	55.68 ± 9.84	58.20 ± 9.84	-0.26	-2.52 (-5.60, 0.56)
Affective Empathy				
Empathic Stress	49.79 ± 9.16	50.08 ± 10.46	-0.03	-0.29 (-3.71, 3.13)
Empathic Joy	58.40 ± 9.20	60.60 ± 9.04	-0.24	-2.20 (-4.34, -0.06)*
**TMMS-24**				
Total	87.76 ± 8.75	91.40 ± 9.94	-0.40	-3.64 (-6.31, -0.97)*
Emotional Attention	29.44 ± 5.45	30.16 ± 5.00	-0.13	-0.72 (-2.21, -0.77)
Emotional Clarity	29.40 ± 4.68	31.16 ± 4.73	-0.37	-1.76 (-3.50, -0.02)*
Emotional Repair	28.92 ± 5.27	30.08 ± 5.70	-0.21	-1.16 (-2.35, 0.36)
**IRI**				
Total	70.60 ± 9.82	72.40 ± 9.75	-0.18	-1.80 (-4.48, 0.88)
Fantasy	19.12 ± 6.07	19.76 ± 6.42	-0.10	-0.64 (-1.81, 0.53)
Perspective-Taking	20.44 ± 4.13	21.08 ± 4.06	-0.16	-0.64 (-1.79, 0.51)
Empathic Concern	20.60 ± 3.62	21.04 ± 3.43	-0.12	-0.44 (-1.52, 0.64)
Personal Distress	10.44 ± 3.63	10.52 ± 4.04	-0.02	-0.08 (-1.52, 1.36)

TECA: Cognitive and Affective Empathy Test; TMMS-24: Trait Meta-Mood Scale; IRI: Interpersonal Reactivity Index

Values are expressed as means ± standard deviation (SD) for pre-post coaching intervention and as mean (95% confidence interval) for within-group (baseline to follow-up) and between-group change scores (at follow-up)

*Significant Student t-test, *P* < 0.05

### Change scores in occupational therapy students group for TECA, TMMS-24 and IRI

In Occupational Therapy Students Group, no significant differences were found for the variables of the TECA (t ≥ 2.00, *p* ≥ 0.052) or the TMMS-24 (t ≥ 0.14, *p* ≥ 0.07). However, the results showed in this group a significant increase in the scores for the Perspective-Taking subscale, which corresponds to the cognitive dimension of the IRI (t = -2.92, *p* = 0.006). The effect size was small (*r* = -0.14). Also, there was a significant decrease in the scores for the Personal Distress subscale, which corresponds to the emotional dimension of the IRI (t = 2.24, *p* = 0.031). The effect size was small (*r* = 0.12). No significant differences were obtained in the rest of variables for this test (t ≥ 0.46, *p* ≥ 0.65) (see Table 5).

**Table 5:** Pre-post coaching intervention differences, change scores in occupational therapy students group (*n* = 39) (95% confidence interval) for TECA, TMMS-24 and IRI

Outcome	Pre-Coaching Intervention	Post-Coaching Intervention	Cohen’s d (Pre/Post)	Score Change
**TECA**				
Total	57.63 ± 11.16	58.16 ± 10.16	-0.05	-0.53 (-2.52, 1.46)
Cognitive Empathy				
Perspective Adoption	55.92 ± 11.95	58.39 ± 10.92	-0.22	-2.47 (-5.28, 0.33)
Emotional Understanding	56.53 ± 10.09	57.10 ± 9.80	-0.06	-0.58 (-3-06, 1.90)
Affective Empathy				
Empathic Stress	52.84 ± 9.34	50.58 ± 9.93	0.23	2.26 (-0.02, 4.55)
Empathic Joy	58.03 ± 8.81	57.68 ± 9.08	0.04	-0.34 (-1.75, 2.44)
**TMMS-24**				
Total	91.47 ± 12.31	89.29 ± 11.37	0.18	2.18 (-1.40, 5.77)
Emotional Attention	32.55 ± 5.26	31.10 ± 4.79	0.29	1.45 (-0.11, 3.07)
Emotional Clarity	29.37 ± 6.16	29.47 ± 5.77	-0.02	-0.10 (-1.67, 1.46)
Emotional Repair	29.55 ± 6.60	28.71 ± 6.47	0.13	0.84 (-0.76, 2.45)
**IRI**				
Total	70.32 ± 11.32	70.55 ± 11.45	-0.02	-0.24 (-2.18, 1.71)
Fantasy	17.87 ± 5.27	18.16 ± 6.65	-0.05	-0.29 (-1.60, 1.02)
Perspective-Taking	19.24 ± 4.70	20.50 ± 4.30	-0.28	-1.26 (-2.14, -0.39)*
Empathic Concern	20.87 ± 3.97	20.66 ± 3.66		0.21 (-0.72, 1.14)
Personal Distress	12.34 ± 4.26	11.24 ± 4.87	0.24	1.10 (0.10, 2.11)*

TECA: Cognitive and Affective Empathy Test; TMMS: Trait Meta-Mood Scale; IRI: Interpersonal Reactivity Index

Values are expressed as means ± standard deviation (SD) for pre-post coaching intervention and as mean (95% confidence interval) for within-group (baseline to follow-up) and between-group change scores (at follow-up)

*Significant Student t-test, *P* < 0.05

## Discussion

The results of this study show an improvement in the cognitive dimension of empathy in nursing students and the affective dimension of empathy of TECA in physiotherapy ones. In addition, total perceived EI end emotional clarity dimensions showed better scores in both nursing/physiotherapy students and in emotional repair (TMMS-24) in nursing degree, at post-intervention. Finally, an overall improvement in cognitive (perspective-taking) and emotional dimension (personal distress) of empathy (IRI) was founded among occupational therapy students after an academic coaching intervention.

Literature about the use of academic coaching in health sciences is still limited in comparison to fields such as sports, music, business and even medicine [10, 27]. To our knowledge, no previous studies have analysed, the effect of academic coaching on self-perception of EI and empathy among students of health sciences in the three disciplines studied herein. The literature review reveals that, at present, researchers are questioning whether the traditional methods of teaching and learning are capable of preparing health sciences students effectively and adequately for their future clinical placements [14, 15, 17, 18]. In this line, we have observed that the scientific evidence proposes numerous alternative approaches to teaching, in addition to coaching, with a view to improving learning outcomes among students of health sciences and other disciplines. Occasionally, however, the results of these approaches are contradictory.

On the one hand, there are studies that demonstrate the effectiveness of these new methodologies, such as conducted by Ward et al. [7] with physiotherapy students. They explored how a new approach to learning, using a virtual cultural simulation experience and guided reflection, significantly improved students’ intrapersonal cultural empathy and their level of satisfaction with the learning experience. In a recent study conducted by Martín et al. [28] with students from a university where classes are taught online, the authors suggested that educational coaching offers an efficient and effective means of helping students to achieve success in their university studies. In the study by Bas-Sarmiento et al. [1], nursing students were given empathy training via methodologies directly related to coaching, such as simulation through role-playing, behaviour assay, flipped classroom and reflective writing. The results of the study indicated that the training was effective in improving the students’ levels of empathy. Similarly, the quasi-experimental prospective study conducted by Fortune et al. [29] also adopted a new teaching methodology, in combination with coaching, to implement a training programme for physiotherapy and occupational therapy students involving the use of motivational interviews. The results showed that the training improved the levels of confidence and empathy in both groups of students. In this line, Mueller et al. [30] analysed the impact of an online evidence-based course on empathy, resilience and work engagement, which was attended by physiotherapy students during their clinical placements. The results of the study suggested that online capacity-building can have a positive impact on empathy, resilience and work engagement among physiotherapy students, and that it could also be applied to other health sciences degrees. Also, the pre-post quasi-experimental study of Romano et al. [31], that assess the impact of a health coaching program in 25 nursing students in Italy, showed statistically significant improvement in the students’ perception of their own stress management skills after the intervention, so the health coaching intervention could improve performance of nursing students [31].

However, other studies do not support the effectiveness of these new methodologies so conclusively. In this line a randomised controlled trial conducted in Germany with students of medicine, measured the improvement in the students’ empathy levels after they had completed a training programme involving simulated patients [9]. Their results were compared with a control group, the experimental group showed significantly higher levels of empathy when they were scored by the patients and experts, but no significant differences were observed between the groups in their self-assessment of rich their attitudes towards empathy [9]. Likewise, an experimental study was conducted with second-year physiotherapy students in which the effectiveness of two educational approaches - self-directed learning and traditional instruction - were compared in terms of the level of knowledge acquisition, optimism, hope and resilience, among other variables. No significant differences were found between the groups for any of these variables, and the authors concluded that more and longer-term research was needed in order to determine whether students benefited from self-directed learning [32].

With regard to the results, we obtained for the TECA, we have not found any published studies that used the same test on a similar sample. The scores for the nursing students were mid-range, in terms of their overall scores as well as those for the different subscales. Physiotherapy and occupational therapy students recorded higher overall scores in TECA’s subscales, with the exception of the Empathic Stress subscale, where the scores were average. However, it should be noted that a high score in the Empathic Stress subscale indicates a certain tendency to become over-involved in the problems of others, consequently, a lower score would indicate a more suitable level of emotional involvement in interpersonal relationships [22].

In the results obtained for the TMMS-24, both the overall scores and those of the subscales demonstrated adequate levels of perceived EI following the intervention, for all three groups (Attention: 22 ≥ x ≤ 35; Clarity and Repair: 24 ≥ x ≤ 35). We observed also a significant improvement in the overall test scores for nursing and physiotherapy students after the intervention. In line with our results, Yoong et al., in 2023 showed that EI improved significantly in nursing students who had participated in a palliative and end-of-life simulation program [32]. However, there were no such changes for the occupational therapy students. In contrast, Polonio-López et al. [34], in which study the TMMS-24 was used to measure the EI of occupational therapy students before and after they carried out their clinical placements, demonstrated that a program centred on “practical training” the students improved their attention to feelings, their emotional clarity and the regulation of their emotions related to EI. In this respect, a number of studies have analysed the relationship between the levels of EI in health sciences students and their ability to pursue a professional career in the future. In this line, Andonian et al. [35] conducted a study with occupational therapy students from 36 universities in the United States. The authors concluded that the students’ levels of EI improved when they were able to undertake clinical placements, which in turn correlated positively with improved communication and intervention skills. Brown et al. [36] examined the question of whether the EI and personality traits of occupational therapy students were predictors of their teamwork skills. The results showed that the variables of emotional reasoning, emotional self-management, emotional management of others and the personality traits of extroversion and emotional stability were significant predictors of the students’ teamwork skills. Similarly, another study [37] explored changes in EI among physiotherapy, occupational therapy and speech therapy students using the Emotional Quotient Inventory 2.0 (EQ-i 2.0), before and after they carried out their clinical placements. The results showed a significant reduction in assertiveness, while the rest of the scores showed no significant increases.

With regard to IRI, if we compare the results we obtained for the health sciences students with the results of the study conducted by Jiang et al. [38] in China with students from other university degree programmes, such as the arts, science and engineering, in which the authors measured empathy using the same scale, we can see that empathy levels as recorded in the IRI are much higher for the health sciences students (as observed in our results) compared to students from other disciplines, where the total average score for the scale was 37.73 points. These findings concur with the study conducted by Holmes et al. [11] which notes that, over the last 30 years, there has been a reduction in the empathy levels of university students in general, although the levels remain higher in health sciences students than in students from other disciplines. The study also highlights the observation that the empathy levels of these students lessen as they progress further into their studies. In this respect, Mueller et al. [30] affirm that academic staff in the physiotherapy field have observed a constant increase in students’ emotional distress and, in general, students of healthcare-related subjects often experience fatigue and a loss of empathy. Moreover, the situation worsens as they progress further into their studies. In the study conducted by Ogino et al. [39] with nursing professionals, the participants obtained a significantly lower score on the Fantasy subscale of the IRI compared to the control group. These lower scores indicated that the nurses had a greater tendency, in comparison to the control group, to adopt a more realistic view, which helped them to care for patients practically and efficiently regardless of their emotional state. In our study, which was conducted with students, the scores for this specific subscale of the IRI were lower for the nursing students than those from other disciplines; moreover, these scores became lower still after the coaching intervention.

Our results for the IRI are also supported by those of the study conducted by Ardenghi et al. [40] with students of medicine, in which the IRI scores are very similar to those recorded in our study and fall within similar ranges. All of these results can help us to highlight the importance of conducting tests and trials to assess empathy and EI, and the importance of taking specific steps to develop these skills, with particular reference (as we explored in our study) to health sciences students prior to the commencement of their clinical practice.

The pilot nature of our study excluded key features of a pivotal clinical trial, including a large sample size, blinding of the intervention and a control group. As clinical implications, it is worth mentioning that adherence to the intervention was good (100%) completed the intervention and surveys; and we speculate that the lack of worsening post-intervention evidences the effectiveness of our training program. In addition, aspects of empathy and emotional intelligence have improved, which seems to indicate that coaching can play an important role in the success of students of health sciences. However, without control group, we can only speculate on what the outcome of the group will be without intervention. So, this study provides preliminary data to design a subsequent clinical trial. Our study design is in line with studies which we have mentioned previously that performance and pre-post study [7, 29, 33, 34] or not include a control group [32, 35–38, 40–42]. Also, in line with our results, a prospective single-arm intervention study conducted in 2019, which evaluated a program of two group and two private training sessions in first-year medical students, obtained good adherence since 37 of 39 (94.9%) completed the protocol [41]. A recent study that evaluated the opinions of different stakeholders (students, faculty members and educational mangers) about the practice of coaching showed that the perception about coaching practices in the three groups were positive and supportive of each other, so their results support the implementation of coaching in nursing education programs [42].

However, other studies have included a control group [20, 30, 31] or have carried out a randomized clinical trial [43]. Coaching is also used in health professionals, in this sense the study by Pollak et al. [20], 2020, with intervention through communication coaching to teach motivational interviews to palliative care clinicians, determined that compared to the control group, the intervention group showed higher motivational interviewing skills scores, higher communication skills, and better burnout scores. Thus, it appears that coaching can improve communication among palliative care clinicians [20]. Also, in this line, in a recent randomized clinical trial Pollak et al., 2023, analysed the effect of a coaching intervention to improve cardiologists’ communication with patients. Their results showed that the skills of expressing empathy and eliciting questions improved after the intervention in the coaching group, which could improve patients’ experience and understanding of the information [43].

Finally, we should also highlight the importance of training the academic staff and professionals who serve as the students’ clinical tutors, as they can directly influence the students’ acquisition of these skills. In our study we have considered up to four control variables that can influence the development of the training program (the training medium, the number of training sessions, the duration of the training program and the demographics of the trainers). However, previous studies highlight the lack of consensus on these variables and therefore the need to carry out future studies where these aspects are analysed in depth [10, 14–16, 20, 25, 26]. They also highlight the need to include and check these variables when implementing a coaching program.

### Limitation

This study has significant limitations. Firstly, we selected a convenience sample of health sciences students from nursing, physiotherapy and occupational therapy degrees, thus our results cannot be generalised to other degree programmes. Secondly, although our study’s pilot nature offers preliminary data regarding coaching efficacy, further studies are required including a large sample size and a control group with clinical trial desing to corroborate and verify our conclusions. Furthermore, because this was not a randomized study with a control group, the effects of coaching intervention cannot be separated from non-specific effects. In this sense, we have also not been able to blind the participants in the study or the coaches who conduct the sessions due to the nature of the intervention, although the coaches were blinded to outcome measures and baseline examination findings. Thirdly there are significant differences regarding the gender of our sample, as there were fewer male participants than female. We were therefore unable to break down the analysis according to gender. However, the proportion of male students in this study is similar to - and representative of - the proportion of male students enrolled in these three degrees at our university. Fourthly the short duration of coaching intervention makes it necessary for future research to evaluate the effects over a longer period, and to extend the follow-up period. Accordingly, caution is needed in extrapolating the results of our study. Consequently, we believe new studies should be conducted that include a higher number of male students and students from different universities. We have considered four control variables that can influence the development of the training program (the training medium, the number of training sessions, the duration of the training program and the demographics of the trainers), however, future studies where analyzing these aspects in depth are necessary. Lastly, there is still no consensus regarding the definition of empathy [44], which makes it a difficult quality to measure. Additionally, we used self-reported questionnaires to empathy and EI, which means the participants’ subjectivity, could affect the results. Also some students may have given socially acceptable responses that are not a true reflection of their empathy skills. However, we have used scales that were available in Spanish and measure empathy from a multi-dimensional perspective by incorporating both emotional and affective aspects, thereby offering a more complex approach to the subject.

## Conclusion

This prospectively designed pilot study provide preliminary efficacy data of a coaching intervention for improving Empathy and EI in students of health science. Main findings show that an academic coaching intervention appears to be an effective modality to improve the cognitive dimension of empathy in nursing students and the affective dimension of empathy in physiotherapy students, as well as, total perceived emotional intelligence and emotional clarity. However, in occupational therapy students the academic coaching intervention produced slight improvements in Perspective-Taking and Personal Distress. Further studies with a larger sample size and allowing a randomized controlled study design will be required.

## Data Availability

The datasets used and/or analysed during the current study are available from the corresponding author on reasonable request.
