# Correction: Implementation of a coaching training for enhancing empathy and emotional intelligence skills in health science students: a prospective study

**DOI:** 10.1186/s12909-024-05168-w

**Published:** 2024-02-23

**Authors:** María Encarnación Aguilar-Ferrándiz, Sonia Toledano-Moreno, Antonio Casas-Barragán, Manuel Albornoz-Cabello, Rosa María Tapia-Haro, María Correa-Rodríguez

**Affiliations:** 1 Department of Physical Therapy, Faculty of Health Sciences, University of Granada (UGR), Instituto de Investigación Biosanitaria ibs.GRANADA. Granada, Ave. de la Ilustración, 60, 18071 Granada, Spain; 2 Department of Physical Therapy, Faculty of Nursing Physiotherapy and Podiatry, University of Sevilla (US), Sevilla, Spain; 3 Department of Nursing, Faculty of Health Sciences, Instituto de Investigación Biosanitaria ibs.GRANADA, University of Granada (UGR), Granada, Spain

**Correction: BMC Medical Education (2024) 24:76**

10.1186/s12909-024-05076-z

Following publication of the original article [1], we have been informed that the given name and family name were swapped.

The original article has been corrected.
